# Dissection of the Mechanism for Compatible and Incompatible Graft Combinations of *Citrus grandis* (L.) Osbeck (‘Hongmian Miyou’)

**DOI:** 10.3390/ijms19020505

**Published:** 2018-02-08

**Authors:** Wen He, Yan Wang, Qing Chen, Bo Sun, Hao-Ru Tang, Dong-Ming Pan, Xiao-Rong Wang

**Affiliations:** 1 College of Horticulture, Fujian Agriculture and Forestry University, Fuzhou 350002, China; hewen0724@gmail.com; 2 College of Horticulture, Sichuan Agricultural University, Chengdu 611130, China; wangyanwxy@163.com (Y.W.); supnovel@gmail.com (Q.C.); sunadam011@163.com (B.S.); htang@sicau.edu.cn (H.-R.T.)

**Keywords:** citrus, graft, anatomy structure, transcriptome, auxin

## Abstract

‘Hongmian miyou’ (*Citrus grandis* L. Osbeck) is mutated from ‘Guanxi miyou’, with a different spongy layer coloration. Trifoliate orange (*Poncirus trifoliata*) is widely used as rootstocks in ‘Guanxi miyou’ grafting, whereas ‘Hongmian miyou’ is incompatible with available trifoliate orange rootstocks. To explore the reasons for the etiolation of leaves of ‘Hongmian miyou’/trifoliate orange, anatomical differences among different graft unions, gene expression profiles, and auxin levels of scion were investigated in this study. A histological assay indicated that there was no significant difference in anatomical structure between the compatible and incompatible combinations. A total of 1950 significant differentially-expressed genes (DEGs) were identified and analyzed. The Kyoto Encyclopedia of Genes and Genomes (KEGG) pathway enrichment analysis revealed that genes involved in carbohydrate metabolism, energy metabolism, amino acid metabolism, and plant hormone signal transduction were significantly enriched. Moreover, the expression of nine genes in the auxin pathway were upregulated and three were downregulated in compatible combinations compared with those in the incompatible group. Further experiments verified that indole-3-acetic acid (IAA) content increases in the compatible graft combination, which suggests that IAA might promote graft compatibility.

## 1. Introduction

**Figure 1:**
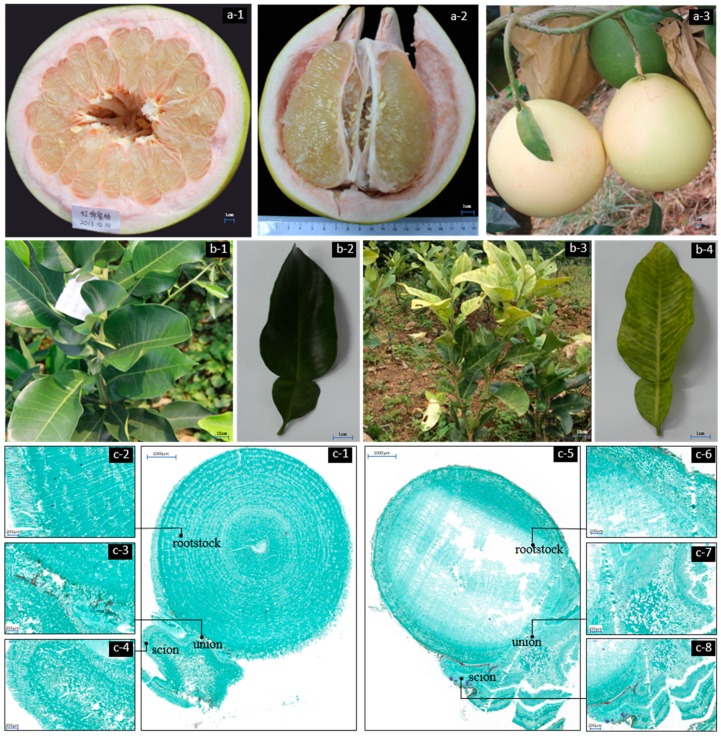
Fruits of ‘Hongmian miyou’, field performance, and anatomical observation of ‘Hongmian miyou’ grafted onto trifoliate orange and fragrant citrus. (**a-1**–**a-3**) Fruits of ‘Hongmian miyou’; (**b-1**,**b-2**) compatibility combination of YS/Cj; (**b-3**,**b-4**) incompatibility combination of Hm/Pt; (**c-1**–**c-4**) anatomical observation of compatibility combination: YS/Cj; and (**c-5**–**c-8**) anatomical observation of incompatibility combination: Hm/Pt. YS/Cj: yellowing seedlings (seedling etiolation of Hm/Pt) grafted to fragrant citrus as a compatible graft combination; Hm/Pt: ‘Hongmian miyou’ grafted to the rootstock of trifoliate orange as an incompatible graft combination.

‘Hongmian miyou’ (*Citrus grandis* L. Osbeck) is mutated from ‘Guanxi miyou’, with a light red color, and accumulates lycopene and β-carotene in the spongy layer (Figure 1a). It has been approved as a new pummelo cultivar by the Fujian Crop Varieties Committee and Sichuan Crop Variety Certification Committee (No. 2011008 and No. 2016016, respectively). Trifoliate orange (*Poncirus trifoliata*) has been widely used as rootstocks in ‘Guanxi miyou’ tree grafting, whereas ‘Hongmian miyou’ is incompatible with available trifoliate orange rootstocks [1]. Until now, no studies have been reported on this incompatible grafting phenomenon. Grafting is an ancient agricultural practice to propagate commercial fruit species and help plants to overcome their limiting factors in growth [2]. However, when grafting involves two different species or genera, a lack of affinity, known as graft incompatibility, may occur [3]. Compatibility/incompatibility between the graft and scion is a complex response including a wide range of anatomical, physiological, and biochemical interactions [4,5,6]. In contrast to the increasing number of studies for the interactions between rootstocks and scions in fruit crops [7,8], the information on the mechanism for graft incompatibility is still limited.

The differentially-expressed genes between compatible and incompatible graft combination can provide key information for the genes related to the incompatibility between the rootstock and scion [9]. A successful grafting begins with the adhesion of rootstock and scion, followed by the formation of callus tissue at the graft interface, and ends with the establishment of vascular connections [10]. Then, plants use their vascular system to distribute nutrients and water throughout the plant body, and also regulate the metabolism of vegetative growth through the transport of phytohormones and genetic information [9,11]. Some incompatibility symptoms occur at the very beginning of grafting, but the presence of some biochemical alterations across the graft union may lead to a slight and delayed incompatibility [6]. Several studies have found that hormones, especially auxin, play significant roles in the regulation of higher plants’ developmental and metabolic traits. Changes of auxin content may be one of the mechanisms involved in physiological effects of grafting [12,13]. Auxin signaling is initiated through the binding of the hormone to the transport inhibitor response l/auxin signaling F-Box protein (TIR1/AFB) or auxin/indole acetic acid (Aux/IAA) protein co-receptors, which results in the degradation of Aux/IAA targeting proteins. The degradation of Aux/IAA proteins allows for the release of Auxin Response Factors (ARFs), which further regulate the expression of auxin-responsive genes. The expression of *Aux/IAAs* and *ARFs* during plant development has been extensively studied in many species [13], yet a lesser extent in grafted plants [7].

In this study, ‘Hongmian miyou’ was grafted to the rootstock of trifoliate orange as an incompatible graft combination (Hm/Pt hereafter) and yellowing seedlings (seedling etiolation of Hm/Pt) were grafted to fragrant citrus as a compatible graft combination (YS/Cj hereafter). The purposes of this study were: (1) to determine the anatomical differences in graft union between Hm/Pt and YS/Cj; (2) to investigate the molecular mechanisms of the effect of incompatible/compatible graft combinations; and (3) to explore the possible role of IAA and its signal transduction pathway in the citrus grafting process.

## 2. Results

### 2.1. Morphological Characteristics and Anatomical Observation

To determine the graft union formation in citrus, the growth status of the graft compatibility and incompatibility was recorded. Field research showed that there was no significant difference in growth until summer shoots matured. Then, the leaves on the summer shoots of Hm/Pt become yellow. At the same time, the chlorophyll content and the photosynthesis activity of the leaves were affected (Figure 1b and Figure S1).

**Figure 2:**
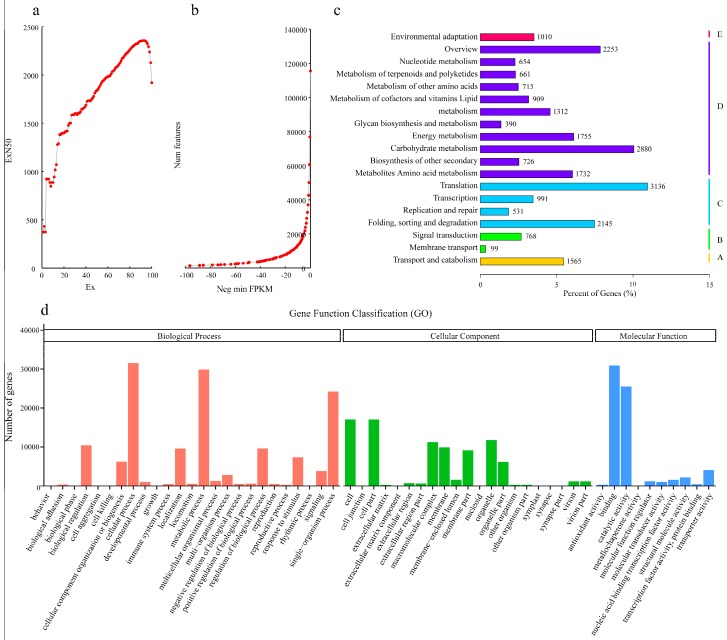
Assembly quality assessment. (**a**) N50 of subset of transcript by decreasing the expression level. Ex denotes the top-most expressed transcripts that represent x% of the data. ExN50 is the length of a transcript, while the total length of transcripts shorter than it reached 50% of the total length of all transcripts in this dataset. (**b**) Transcript count with a threshold of negative minimum FPKM (fragments per kilobase of the exon model per million mapped reads) value. (**c**) The classification of the Kyoto Encyclopedia of Genes and Genomes (KEGG) pathways. (**d**) The classification of the Gene Ontology (GO) pathways.

**Figure 3:**
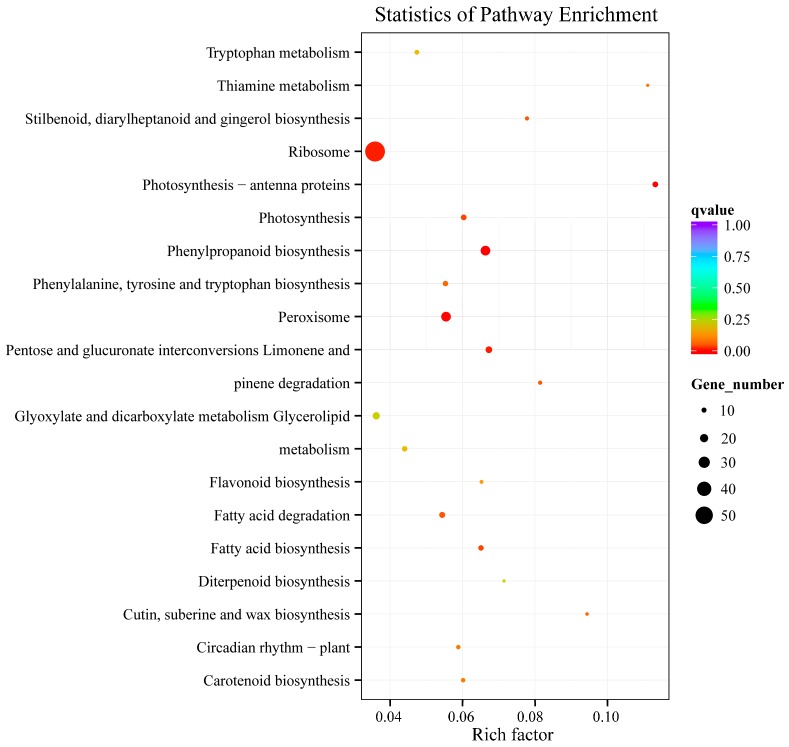
Kyoto encyclopedia of genes and genomes (KEGG) enrichment analysis of the differentially-expressed genes (DEGs) between compatible/ incompatible combinations.

A histological study of the graft interface will provide the basic information about the graft union formation. According to the histological observation, there were no differences in the anatomical structure between compatible and incompatible combinations (Figure 1c). The graft interface was continuous, and the parenchymatous cells close to the graft interface proliferated and adhered to the opposing tissue (Figure 1(c-3,c-7)). The stem cell-like tissue differentiated and gave rise to new vascular tissues, which connected the xylem and phloem between the scion and rootstock. Based on the results obtained from the field performance and anatomical observation, the compatible graft showed higher survival rate than that of the incompatible one but had the same healing process.

### 2.2. Overview of the RNA Sequencing and Classification of Enriched GO and KEGG Terms

In this experiment, six libraries from Hm/Pt and YS/Cj grafting combinations, which represented graft incompatibility and compatibility combinations, were constructed. One tissue from each graft combination was prepared in triplicate. RNA-Seq technology was used to profile the leaf petioles transcriptome on the Illumina Hiseq™ 4000 platform (Illumina Inc., Hayward, CA, USA). After removing sequencing adaptors and low quality data, we obtained more than 278 million clean short reads comprising 41.84 Gb of sequence data. Detail information is shown in Table 1. The sequence generated from Trinity software [14] was used as the reference transcriptome. We generated 269,188 transcripts (N50: 1310) with a mean length of 672 bp. CD-HIT (Cluster Database at High Identity with Tolerance) clustering resulted in 115,504 unigenes (N50: 1922) with a mean length of 1200 bp.

The contig N50 value, defined as the maximum length whereby at least 50% of the total assembled sequence resides in contigs of at least that length, is a commonly used metric for evaluating the contiguity of a genome assembly, while ExN50 has been implemented in the Trinity package [15] and recommended by Haas [16]. The profile of ExN50 vs. N50 value can be useful indicators as to the overall quality of the assembly. Ex and ExN50 have been used to analyze transcriptome data [17]. Taking expression values into consideration, we recalculated the N50 value after eliminating low-expression contigs (Figure 2a), and then plotted the expression value distribution pattern (Figure 2b). Ex in Figure 2a revealed a subset of top x% highly expressed transcripts. The ExN50 reached the maximum length at E92 and E93, showing that 799,150 transcripts were in the top 95% expression subset with the minimum FPKM (fragments per kilobase of the exon model per million mapped reads) of 49.93. Thus, we proposed that most of the extremely high expression transcripts in citrus leaf petioles ranged from 600 to 1000 bp. As shown in Figure 2b, the transcript number decreased to 10,047 after eliminating transcripts with low FPKM values (below 5). All the raw data were deposited into NCBI (National Center for Biotechnology Information) Sequence Reads Archive (SRA) with accession numbers SAMN07640959, SAMN07640960, SAMN07640961, SAMN07640962, SAMN07640963, and SAMN07640964.

We also assigned Gene Ontology (GO) terms to citrus unigenes. A total of 87,509 unigenes (75.76%) could be assigned into at least one GO term, and detailed information on the classification of GO is listed in Figure 2d. Within the biological process category, the most highly represented terms were ‘cellular process’ and ‘metabolic process’. Within the molecular function category, ‘binding’ and ‘catalytic activity’ were the two most abundant terms. The most abundant terms within the cellular component category were ‘Organelle’, ‘cell’, and ‘cell part’.

To further reveal the involvement of metabolic pathways in compatibility/incompatibility grafted combinations, we predicted the KEGG pathways represented by all assembled unigenes. A total of 29,930 unigenes were assigned into 130 signaling and metabolic pathways, including pathways related to cellular processes, environmental information processing, genetic information processing, metabolism, and organismal systems (Figure 2c). Interestingly, the highest proportion of genes were those in KEGG pathways related to metabolic pathways, such as carbohydrate metabolism (2880 unigenes), energy metabolism (1755 unigenes), and amino acid metabolism (1732 unigenes).

### 2.3. Analysis of Differentially-Expressed Genes

Read-mapping data was used to calculate FPKM values, which were normalized indicators for comparing the transcript levels of each unigene between different samples. A total of 1950 significant differentially-expressed genes (DEGs) were identified using a criterion of two-fold change and *p_adj_* < 0.05 (Figure S2). A total of 1079 genes were significantly upregulated and 871 genes were significantly downregulated in YS/Cj, respectively.

Further KEGG enrichment analysis (Figure 3) showed that the differentially-expressed genes were mainly enriched in ribosome, peroxisome, phenylpropanoid biosynthesis, and glyoxylate and dicarboxylate metabolism. Notably, photosynthesis and carotenoid biosynthesis were also enriched. Twelve genes involved in photosynthesis were identified as being differentially-regulated in the two combinations. These genes included Psb-like protein (Cluster-25005.37802, Cluster-28820.3, Cluster-25005.44652, Cluster-25005.29670), Photosystem I reaction center subunit (Cluster-25005.39964, Cluster-25005.36564, Cluster-25005.47754, Cluster-25005.36103, Cluster-25005.44296, Cluster-25005.37563), and photosynthetic electron transport protein (Cluster-25005.38785, Cluster-24560.2). Four chlorophyllase protein coding genes (Cluster-25005.6580, Cluster-25005.53630, Cluster-25005.6580, Cluster-25005.53630) were significantly upregulated and one gene (Cluster-25005.1330) was downregulated in YS/Cj. Meanwhile, the expression levels of seven carotenoid synthesis genes (Cluster-23083.0, Cluster-25005.57495, Cluster-25005.21134, Cluster-25005.66741, Cluster-25005.66740, Cluster-25005.38211, Cluster-25005.45075) were significantly decreased. However, one gene involving carotenoid pathway (Cluster-25005.14249) was upregulated in Hm/Pt.

### 2.4. qRT-PCR Validation of DEGs

**Figure 4:**
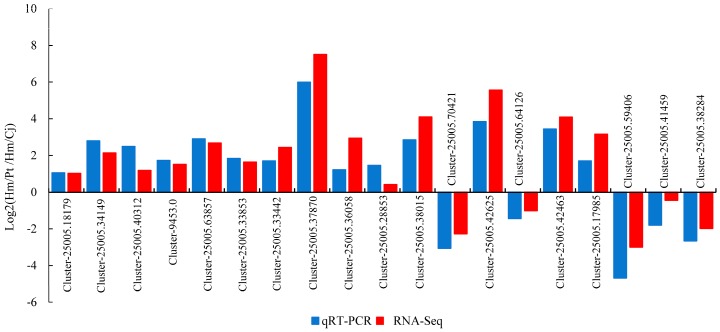
Expression pattern of 19 selected genes obtained by RNA-seq and real-time quantitative PCR (qRT-PCR) methods.

To verify the reliability and accuracy of transcriptome data, 19 DEGs were randomly selected to analyze their expression profiles by real-time quantitative PCR (qRT-PCR). *β-tubulin* was selected for internal controls. The qRT-PCR expression profiles of 19 DEGs were similar to those obtained through high-throughput sequencing (Figure 4). The correlation between qRT-PCR and RNA-Seq was measured by scatter-plotting log2-fold changes, which showed a positive correlation coefficient (Pearson coefficient *R*^2^ = 0.867). These results confirmed the reliability of the genome-wide transcriptome profiling analysis.

### 2.5. Auxin Signal Pathway and Response of Auxin Level

**Figure 5:**
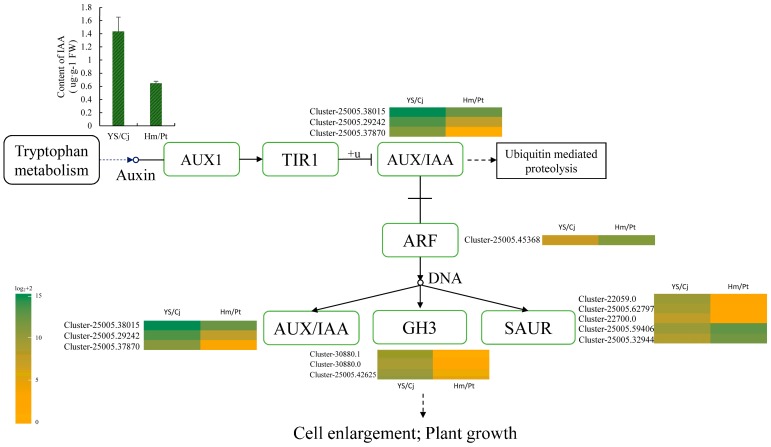
Overview of auxin pathways and their expression profiles and content of indole-3-acetic acid (IAA) in different graft combinations. The value of log_2_(readcount) + 2 was represented using the depth of color, with sea-green representing high expression and orange representing low expression. Note: AUX1 represent auxin resistant 1; TIR1 represent transport inhibitor response l; AUX/IAA represent auxin/indole acetic acid; ARF represent auxin response factors; GH3 represent gretchen hagen 3; SAUR represent small auxin upregulated RNA; Arrows with solid and dashed lines represent the related pathway and the abbreviated pathway, respectively.

Since auxin biosynthesis pathway genes were significantly enriched in the DEG enrichment analysis, the expression of all genes in auxin signaling pathways were analyzed to reveal the involvements of this important hormonal signaling pathways in the graft process of citrus. Comparison of the transcript abundances of genes for auxin transport, metabolism, the signaling pathway, and downstream-induced genes revealed a consistent response during the graft process (Figure 5). The plant hormone auxin, critical for plant growth and development processes, plays its regulatory role mainly by inducing expression of early auxin response genes including *Aux/IAA*, *GH3*, and *SAUR*. Three *Aux/IAA* unigenes (Cluster-25005.38015, Cluster-25005.29242, Cluster-25005.37870) and three *GH3* unigenes (Cluster-30880.1, Cluster-30880.0, Cluster-25005.42625) were upregulated in YS/Cj. For the *SAUR* gene family, most unigenes were induced and three unigenes (Cluster-22059.0, Cluster-25005.62797, Cluster-22700.0) were upregulated. Two (Cluster-25005.59406, Cluster-25005.32944) were downregulated in YS/Cj. Auxin response factors (ARFs), together with auxin/indole acid proteins (Aux/IAAs), are transcription factors that play key roles in regulating auxin-responsive transcription in plants. Only one *ARF*-encoding gene (Cluster-25005.45368) was identified as being down-regulated in YS/Cj.

To confirm the results from transcriptome analysis, we compared the IAA content in leaves between the compatible and the incompatible combinations (Figure 5). The IAA concentration in the compatible combination was significantly higher than that in the incompatible combination (*p* < 0.05). This result was consistent with the gene expression patterns, indicating that IAA might promote the graft compatibility.

## 3. Discussion

### 3.1. Formation of the Stock/Scion Union

The success of grafting primarily depends on the compatibility of the graft union to enable rapid development of vascular connections between the rootstock and the scion [18], which, in turn, will allow quick resumption of the growth of both the root and the canopy [19]. In the grafted plants, vascular regeneration is a complex process that includes structural differentiation of the parenchymatous tissue from both sides of the graft union into xylem and phloem [18,20]. The sequence of structural events occurring during the healing of the graft union in woody and herbaceous plants has been reviewed by several authors [21]. Yin et al. [22] have proposed six major events: (1) wound-induced response; (2) clean-up of cell debris; (3) cellular communication; (4) auxin accumulation and responses; (5) cell division and differentiation; and (6) vascular reconnection. It is now known that some mRNA signal changes just after 24 h of grafting, and that auxins increase at the union and stimulate cell division and differentiation after 48 h. On the third day, transport is already functioning across the graft union [22,23]. This is an indication that new xylem and phloem formation follow the same pattern described by Dengler [24] in normal shoots and roots. In this study, there were no significant differences in anatomical structure between compatible and incompatible combinations of ‘Hongmian miyou’ (Figure 1). Material transport or signaling can also lead to incompatibility. Take *Prunus armeniaca* as an example: the phenylpropanoid pathway has been identified as one of those responsible for physiological failure in stock-scion combinations [25].

### 3.2. Metabolic Pathways

A visible symptom of Hm/Pt trees is leaf chlorosis in young leaves, which is mainly due to the reduction of chlorophyll content. The present study showed that the expression of the chlorophyll degradation gene, *CLH*, was significantly lower in YS/Cj than that in Hm/Pt. This could explain why the color in the leaves from YS/Cj was lighter than from Hm/Pt. Carotenoids, including carotenes and xanthophylls, are also important photosynthetic pigments [26]. Carotenoids are physiologically relevant because of their roles in photosynthesis and participation in light harvesting, energy transfer, quenching, and photoprotection [27,28]. Leaf chlorosis and reduction of carotenoids will directly lead to a decrease in photosynthesis [29]. In this study, the expression levels of photosynthesis-related genes were significantly upregulated in YS/Cj. These results suggested that graft incompatibility decreased photosynthesis ability through downregulating the expression of photosynthesis-related genes. On the other hand, the carotenoid synthesis-related genes were also significantly downregulated in Hm/Pt. Therefore, the significant decrease in carotenoid synthesis-related gene and photosynthesis-related genes in Hm/Pt at least partially explained the reason for the lower photosynthesis ability (Figure S1) of Hm/Pt than that of YS/Cj.

### 3.3. The Role of Hormones

The plant hormone auxin is critical for plant growth and development processes [30]. Its role in the incompatible Hm/Pt combination was investigated. Auxin signaling is initiated through binding of the hormone to the transport inhibitor response 1/auxin signaling F-Box protein (TIR1/AFB) and auxin/indole acetic acid (Aux/IAA) protein co-receptors, which results in degradation of the targeted Aux/IAA proteins. The degradation of Aux/IAA proteins allows the release of auxin response factors (ARFs), further regulating the expression of the auxin-response gene. The expression of *Aux/IAAs* and *ARFs* during the growth period of grafted plants has been extensively studied in many species [7]. In this study, a large number of *ARF* and *Aux*/*IAA* genes were identified. The changes in their expression patterns indicated a key role of auxin signaling in control graft union healing and graft compatibility. In model plants, a block in auxin transport at the grafted junction could cause auxin accumulation in the scion, increasing xylem differentiation [31]. In addition, the expression of three *GH3* genes (Cluster-30880.1, Cluster-30880.0, Cluster-25005.42625) also showed responses to grafting in citrus. In grape, the different kinetic of IAA-Asp accumulation at the grafting stages was associated with the expression pattern of the *GH3* gene, named *VviGH3-21* [13]. This shift in IAA-Asp accumulation may play an important role in the grafted plant growth.

## 4. Materials and Methods

### 4.1. Plant Materials

Plant materials were planted in the orchard of Sichuan Agriculture University, Chengdu, China. ‘Hongmian miyou’ (Hm hereafter) was selected as scions. Two-year-old ‘trifoliate orange’ (Pt hereafter) and ‘fragrant citrus’ (Cj hereafter) were used as rootstocks. Then, there were two experimental groups, Hm grafting onto Pt as an incompatible graft combination (Hm/Pt) and yellowing seedling grafting onto Cj as a compatible graft combination (YS/Cj). All materials were grafted by using the splice grafting method. More than thirty plants per graft combination were used for this study. Plant materials used in this study included the graft union, leaves, and petioles. To evaluate the contact surface, the graft union from three plants (three replicates) in each graft combination were collected. Mature leaves in summer shoots were selected for the measurements of chlorophyll content, photosynthetic characteristics, and endogenous phytohormones. All these samples were collected six months after grafting. Graft union fixed in FAA solution (formalin:acetic acid:70% alcohol = 1:1:16) and other samples were frozen in liquid nitrogen and stored at −80 °C until use.

### 4.2. Anatomical Structure Observation

Samples were softened in glycerol:alcohol mixture (1:1) more than one week, then dehydrated in an ethanol series (15%, 30%, 50%, 70%, and 95%) for 90 min each. After infiltration in Safranin and Fast green FCF, decoloration was carried out using dimethylbenzene. Specimens were embedded in paraffin at the end. Samples were sectioned at a thickness of 10 μm on a sliding microtome. Finally, the paraffin sections were observed by using the Nikon Eclipse CI (Nikon, Tokyo, Japan) and photographed with a Nikon DS-U3 (Nikon, Tokyo, Japan).

### 4.3. RNA Extraction, Library Construction, and Sequencing

Equal, but small, amounts of Hm leaf petioles from three plants were pooled together as one replicate. Three independent replicates were carried out for RNA extraction. RNA concentration was measured using a Qubit^®^ RNA assay kit in a Qubit^®^ 2.0 fluorometer (Life Technologies, Carlsbad, CA, USA) following the manufacturer’s instructions. RNA quality was verified using an RNA Nano 6000 assay kit of the Agilent Bioanalyzer 2100 system (Agilent Technologies, Santa Clara, CA, USA) and monitored on 1% agarose gels. A total amount of 3 μg RNA per sample was used as input materials. Sequencing libraries were generated using the NEBNext Ultra Directional RNA library prep kit from Illumina (NEB, Ipswich, MA, USA) following manufacturer’s recommendations.

The cDNA library from each sample was sequenced on an Illumina Hiseq^TM^ 4000 platform (Illumina Inc., Hayward, CA, USA) and paired-end reads were generated.

### 4.4. De Novo Assembly and Gene Expression Analysis

Before sequence assembly, the adapter sequences and low quality reads were removed from the raw reads. At the same time, Q20, Q30, GC-content, and sequence duplication levels of the clean data were calculated. All the downstream analyses were based on clean data with high quality. Trinity (v2.2.0) [14] was utilized with the default parameters except that the minimum contig length was set to 200 bp, reads were first normalized with a maximum coverage of 50 before putting in the assembly pipeline, and the kmer coverage was set to be at the minimum level of 2. Redundancy in the de novo transcriptome was minimized with CD-HIT-EST (v4.6.4) [32] using an identity cutoff at 0.99. All reads in each sample were mapped back to the transcriptome using bowtie2 [33] (default parameters, but end-to-end, allowing two bases of mispairing and multiple hits ≤20) and then RSEM [34] was used to estimate the expression values for each transcript as FPKM. The sets of DEGs were identified using the DEGseq R package with a *q* value < 0.005 and |log_2_ (fold-change)| > 1.0.

### 4.5. Gene Annotation

Gene function was annotated based on the following databases: Nr (NCBI non-redundant protein sequences); Nt (NCBI non-redundant nucleotide sequences); Pfam (Protein family) [35]; KOG/COG (Clusters of Orthologous Groups of proteins) [36]; Swiss-Prot (a manually annotated and reviewed protein sequence database) [37]; KO (KEGG Ortholog database) [38]; and GO (Gene Ontology) [39] using the local blast2go package.

### 4.6. qRT-PCR Validation

The qRT-PCR primer sequences were designed using Primer Premier 5 software (Premier Biosoft International, Palo Alto, CA, USA) and listed in Table S1. The qRT-PCR was performed in a 10 μL reaction volume using the Thunderbird TM SYBR qPCR Mix (TOYOBO, Osaka, JAPAN) on a CFX96 Touch™ Real-Time PCR detection system (Bio-Rad, Hercules, CA, USA). Conditions for amplification consisted of an initial incubation at 50 °C for 2 min and 95 °C on at 50 °C for 2 min and denaturation at 95 °C for 10 min, followed by 40 cycles at 95 °C for 15 s and 60 °C for 60 s [40]. The citrus *β-Tubulin* gene was used to calibrate the relative fold-differences based on comparative cycle threshold (2^−ΔΔ*C*t^) values.

### 4.7. Determination of the Chlorophyll Content; Photosynthetic Characteristics

Photosynthetic and environmental parameters were calculated by a portable photosynthetic analyzer (Li-6400, Lincoln, NE, USA). Then the leaves were collected and suspended in 10 mL of 80% acetone and kept overnight in darkness. The chlorophyll content of graft combinations was measured by a spectrophotometer [6].

### 4.8. Determination of the Endogenous Phytohormones

In each graft combination, Hm leaves from ten plants were pooled together as one sample. HPLC (High Performance Liquid Chromatography) was used to determine the concentrations of indole-3-acetic acid (IAA) of the leaves of compatible/incompatible combinations. Abundant cold methanol (80%, *v*/*v*) was used to extract IAA. Crude extract was condensed by vacuum evaporating and hormones were re-extracted by ethyl acetate at pH 3.0 [41]. Then condensed IAA extracts were purified by Sep-Pak C18 columns. The purified extracts were dissolved in 50% aqueous MeCN containing 0.2% CH_3_COOH. Ten microliters (10 μL) of samples were applied to the HPLC analysis. The conditions of HPLC (1260 Infinity, Agilent Technologies, Santa Clara, CA, USA) were as follows: C18 column (Ultra Aqueous C18 150 mm × 4.6 mm, Restek, Bellefonte, PA, USA); detector wavelength: 254 nm; temperature: 35 °C; and flow rate: 1.0 mL·min^−1^.

## 5. Conclusions

The present study indicated that seeding etiolation cannot ascribe to a familiar formation of the stock/scion union. Many DEGs were identified in citrus during the grafted plants’ growth. Transcription dynamics of grafting response genes and their related major biological functions were grouped into different GO and KEGG categories. Furthermore, the auxin content level and the expression of 12 DEGs related to auxin signaling pathways were analyzed in ‘Hongmian miyou’ and the results showed that auxin might promote the graft compatibility. Hence, this study would provide basic information to help us to understand the role of auxin in graft incompatibility.

## Figures and Tables

**Table 1:** Statistics of sequencing data of the six libraries.

Sample ID	Raw Reads	Clean Reads	Clean Bases (G)	GC Content (%)	Q30 Ratio (%)	Mapped Ratio (%)
YS/Cj_1	50,901,804	49,522,214	7.43	43.81	92.77	83.91
YS/Cj_2	54,064,700	51,584,962	7.74	43.29	92.94	79.9
YS/Cj_3	45,805,274	44,227,228	6.63	43.7	90.04	81.72
Hm/Pt_1	52,696,450	51,269,554	7.69	43.86	92.91	83.29
Hm/Pt_2	44,024,194	41,907,724	6.29	43.17	93.42	79.18
Hm/Pt_3	42,844,292	40,428,286	6.06	43.49	91.52	81.3

## Supplementary Materials

The following are available online at http://www.mdpi.com/1422-0067/19/2/505/s1.
