# Supplementary material for: Dissection of the Mechanism for Compatible and Incompatible Graft Combinations of Citrus grandis (L.) Osbeck (‘Hongmian Miyou’)
Source: Int J Mol Sci. 2018 Feb 8;19(2):505. doi: 10.3390/ijms19020505 (PMC5855727; doi:10.3390/ijms19020505)

Figure S1. Chlorophyll contents and photosynthetic characteristics of different graft combinations

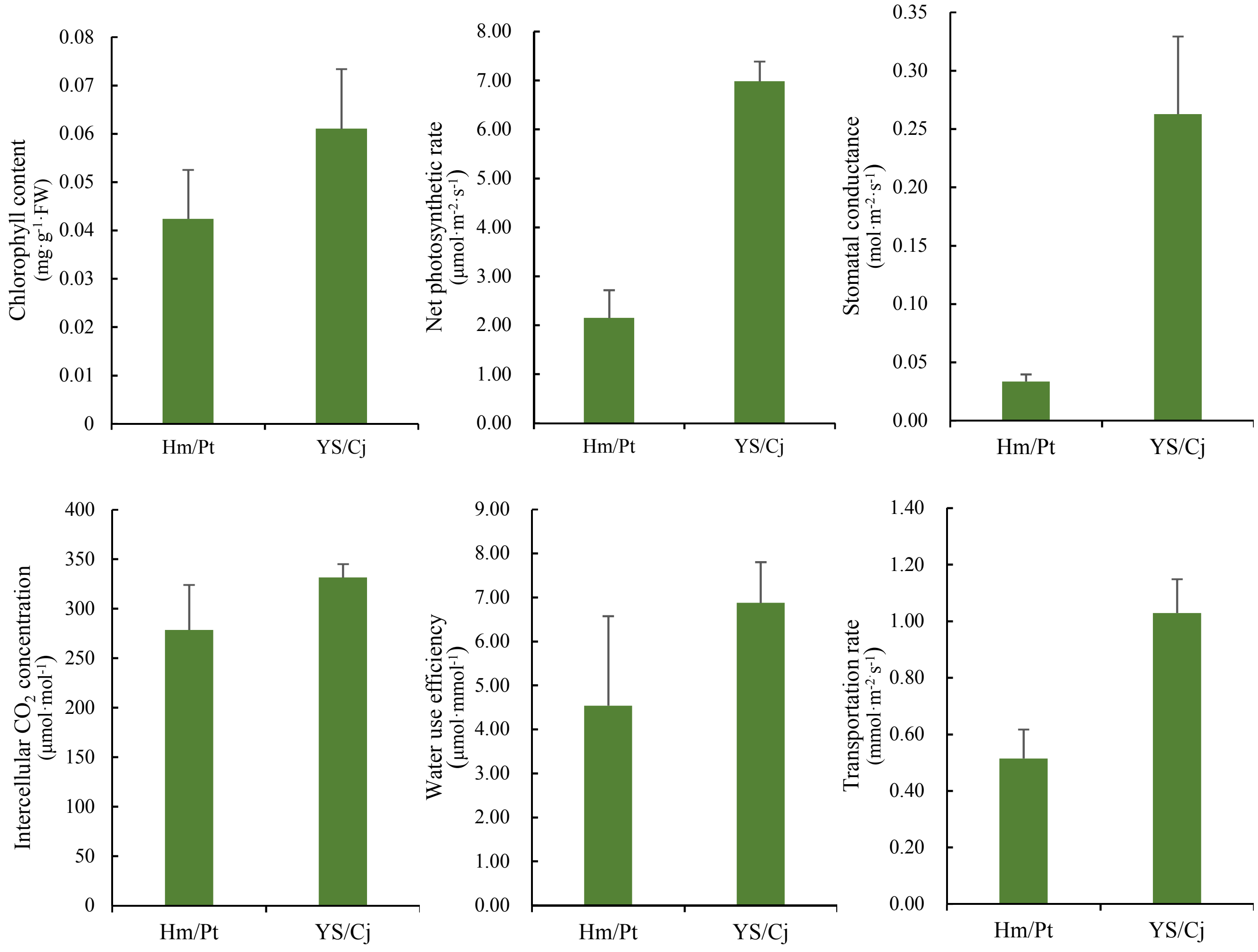

Table S1 Details of selected genes and the sequence of primers

Code	Gene id	Primer Forward	Primer Reverse
1	Cluster-25005.18179	GCAGATGGAAGTGTTAGC	AATGTGGAAGCCTCAGAA
2	Cluster-25005.34149	GGCGATTAACTGGATTACG	GGAAGCACTGGAGGAATA
3	Cluster-25005.47497	TGCTATCTTCTATACCGTTCTCT	GTCTTGCTGCGATTGCTA
4	Cluster-25005.40312	CTTCGTCTTCTGAATTACAT	AGCCAAGAACAATAACATTA
5	Cluster-9453.0	TCATAAGTAGGTTCATATTCAG	CGCCATATCTAAGGAAGA
6	Cluster-25005.63857	TTGCCGTTACCACTATTAG	GGAACATCAACAGAGACTT
7	Cluster-25005.33853	CTTATAGGAACACCGAGGAT	CAGATGGATAGAGCAGGAT
8	Cluster-25005.33442	GAACATCTCCGACTAACAT	GATTAAGTGAGGCATTGAAG
9	Cluster-25005.37870	ATTCGGCTGTTAGTTATTGG	GGTGATTGGATGCTTGTG
10	Cluster-25005.36058	TCATCGTCTCCAGCATTA	GTTAGAGCCAACAATAATAAGC
11	Cluster-25005.28853	TTATGAAGAGCACTGATG	GGATGAATACCAACAATATG
12	Cluster-25005.38015	TGTTGTTGGAAGGAGGATT	CTGTGAGACTGCCGTAAT
13	Cluster-25005.70421	GTTCGCTACATACCAGGAA	CGGCAATCAACCAATCTC
14	Cluster-25005.42625	GATACTGCTTCTCTTCTC	TACTTCTGTCTTCCTTCT
15	Cluster-25005.64126	TAAGCCACCTAAGATTGT	GCAGCATACTTCTATCAC
16	Cluster-25005.42463	AGGCTTGAACATCTCTTG	TCTTGCTTCTTGCTTCTT
17	Cluster-25005.17985	TGCCTGGTATTGTTCAAG	ACTTCTGTTGCTGTCATAG
18	Cluster-25005.10880	ATTAAGGAAGAGCCACAT	GCAACTGATGATACAATGA
19	Cluster-25005.25681	GCATCAATCAATCACTATATG	CTAACTAGCAGACTGTAGA
20	Cluster-25005.59406	CGAATTTACAAGAGAGAAT	ATTATTGAGTTGGTTAGTG
21	Cluster-25005.41459	TTATTGGCAGTGGATGAT	AGAAGTGGTTGATTGGAA
22	Cluster-25005.38284	CACCGATTCCCGAAACAA	GTATGACTGATGATGACGAACA
23	Cluster-25005.32944	GCCTCATTGTGTTACTCA	GCACTTTGAATCCTCTGA
Reference	*β-Tubulin*	ACATCCCGCCTAAGGGTCTG	TTCCTCCGAAACATAGCCGTA

Figure S2. Hcluster heatmap.detail

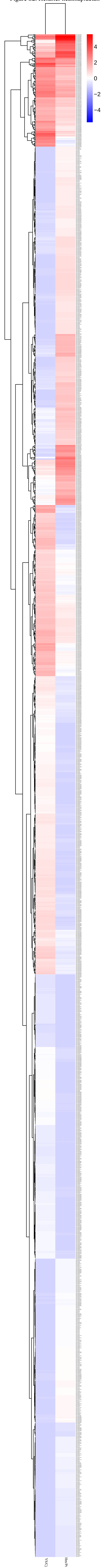

Supplement: Supplementary file 1 [file ijms-19-00505-s001.pdf]
